# Distinct Hypoxemic Profiles of Obstructive Sleep Apnea in Southern Italy: The Living with OSA and CPAP Study

**DOI:** 10.3390/jcm15010206

**Published:** 2025-12-27

**Authors:** Emanuela Resta, Valentina Gnoni, Peter Cistulli, Ivana Rosenzweig, Alessia D’Ambrosio, Preethymol Peter, Vito Antonio Falcone, Vitaliano Nicola Quaranta, Roberto Sabato, Maurizio Domenico Toraldo, Antonio Laricchiuta, Alberto Capozzolo, Elena Capozza, Carla Santomasi, Elisabetta Di Perna, Valeria Dalena, Giuseppe Ricco, Anna Rita Tusino, Simone Sorangelo, Daniela Margiotta, Mariafrancesca Grimaldi, Terence Campanino, Giuseppe Mansueto, Angela Pinto, Giulia Scioscia, Giovanna Elisiana Carpagnano, Annalisa Carlucci, Maria Pia Foschino Barbaro, Donato Lacedonia, Onofrio Resta, Giancarlo Logroscino, Pasquale Tondo

**Affiliations:** 1 Department of Methods and Models for Economics, Territory and Finance, Sapienza University of Rome, 00185 Rome, Italy; 2 Center for Neurodegenerative Diseases and the Aging Brain, University of Bari Aldo Moro at Pia Fondazione “Cardinale Giovanni Panico”, 73039 Tricase, Italy; 3 Charles Perkins Centre and Faculty of Medicine and Health, University of Sydney, Sydney 2006, Australia; 4 King’s College London Institute of Psychiatry Psychology & Neuroscience, London SE5 8AF, UK; 5 Pulmonology Unit, Universo Salute Opera Don Uva, 76011 Bisceglie, Italy; 6 Department of Innovation Engineering, University of Salento, 73100 Lecce, Italy; 7 Pulmonology Unit, San Paolo Hospital, 70121 Bari, Italy; 8 Pulmonology Unit, “Aldo Moro” University of Bari, 70121 Bari, Italy; 9 Respiratory and Intensive Care Unit (RICU), Foggia Polyclinic University Hospital, 71122 Foggia, Italy; 10 Pulmonology Unit, San Cesario Hospital, 73100 Lecce, Italy; 11 Pulmonology Unit, Hospital of Putignano, 70017 Putignano, Italy; 12 Pulmonology Unit, Hospital of Terlizzi, 70038 Terlizzi, Italy; 13 Pulmonology Unit, “Teresa Masselli Mascia” Hospital, 71016 San Severo, Italy; 14 Pulmonology Unit, Hospital of Barletta, 76121 Barletta, Italy; 15 Pulmonology Unit, Hospital of Ostuni, 72017 Ostuni, Italy; 16 Pulmonology Unit, IRCCS Humanitas Research Hospital, 20089 Rozzano, Italy; 17 Department of Medical and Surgical Sciences, University of Foggia, 71122 Foggia, Italy; 18 Pulmonology Unit, Hospital of Trani, 76125 Trani, Italy; 19 Pulmonology Unit, Vito Fazzi Hospital, University of Salento, 73100 Lecce, Italy; 20 Pulmonology Unit, Universo Salute Opera Don Uva, 71122 Foggia, Italy; 21 ASL, 70126 Bari, Italy

**Keywords:** cluster analysis, hypoxemia, phenotypes, sleep apnea

## Abstract

**Background**: Obstructive sleep apnea (OSA) is a heterogeneous disorder associated with substantial cardiometabolic and neurocognitive morbidity. Although the apnea–hypopnea index (AHI) remains the conventional measure of OSA severity, it only partially reflects the underlying pathophysiological complexity. Growing evidence indicates that nocturnal hypoxemia may be a more powerful marker of adverse outcomes than event frequency alone. Therefore, this study aimed to identify distinct OSA phenotypes based on oximetry-derived features and to assess whether these profiles offer additional clinical insight beyond traditional AHI-based classification. **Methods**: This multicenter retrospective study, part of the Living with OSA and CPAP: The Apulia Region Experience project, included 1386 adults diagnosed with OSA across 15 sleep centers in Southern Italy. Standardized clinical, anthropometric, and polysomnographic (PSG) data were collected. Hierarchical clustering analysis was performed based on PSG oximetry-derived variables. Resulting clusters were compared across demographic, clinical, hypoxemic, and therapeutic features. **Results**: Three reproducible clusters emerged. Cluster 1 (mild–non-obese) included younger, leaner patients with lower AHI (22.9 ± 10.5 events·h^−1^), minimal desaturation (T90 5.6 ± 7.6%), and limited comorbidities. Cluster 2 (severe–obese–hypoxemic) represented the most critical phenotype, characterized by marked obesity (BMI 39.2 ± 8.2 kg·m^−2^), severe OSA (AHI 74.9 ± 17.9 events·h^−1^), profound nocturnal hypoxemia (T90 51.5 ± 28.2%), and a high prevalence of metabolic disorders (76%), requiring higher CPAP pressures and frequent oxygen supplementation. Cluster 3 (older–comorbid) comprised older males (63.7 ± 11.8 years) with moderate-to-severe OSA (AHI 44.8 ± 15.2 events·h^−1^) and multiple cardiometabolic comorbidities. **Conclusions**: Oximetry-derived variables identify distinct and clinically meaningful OSA phenotypes that extend beyond traditional AHI-based classification. Recognizing hypoxemia-driven subtypes could improve risk stratification and enable more personalized management strategies in clinical practice.

## 1. Introduction

Obstructive sleep apnea (OSA) is a highly prevalent and heterogeneous sleep disorder [1] characterized by recurrent episodes of partial or complete upper airway collapse during sleep. These events lead to intermittent hypoxemia, sleep fragmentation, and surges in sympathetic activity, promoting oxidative stress, systemic inflammation, and endothelial dysfunction [2,3,4]. Through these mechanisms, OSA contributes to the development and progression of cardiovascular, metabolic, and neurocognitive comorbidities, resulting in increased morbidity and mortality compared with the general population [5].

The apnea–hypopnea index (AHI) has long been the cornerstone for defining OSA severity and guiding clinical decisions. However, several studies have shown that AHI alone insufficiently reflects the disease’s complexity and fails to predict adverse outcomes across individuals [6,7]. Indeed, patients with similar AHI values may exhibit widely variable clinical manifestations and risk profiles, highlighting the need to integrate additional physiological metrics. Among these, the degree and duration of nocturnal oxygen desaturation have emerged as key indicators of OSA-related burden [8,9]. Nocturnal hypoxemia, quantified by indices such as oxygen desaturation index (ODI), cumulative time spent below 90% oxygen saturation during sleep (T90), or composite “hypoxic burden”, has been associated with cardiovascular events, metabolic dysfunction, cancer, and all-cause mortality, even after adjustment for AHI [9,10,11].

Beyond its prognostic value, nocturnal hypoxemia may delineate specific OSA subtypes differing in age, comorbidity patterns, and response to therapy. Data-driven approaches, including unsupervised clustering and machine learning, have recently been applied to disentangle this heterogeneity and identify discrete phenotypes within OSA populations [12,13]. These methodologies have revealed that hypoxemia-related traits, more than traditional polysomnographic parameters, are strongly linked to long-term outcomes and may support a precision medicine approach for disease management [14]. Nevertheless, multicenter studies integrating clinical, functional, and nocturnal oximetric variables to characterize hypoxemic phenotypes in real-world OSA populations are still limited.

In this context, the present study fills a critical gap by utilising a large multicentre cohort to verify whether variables derived from nocturnal oximetry can more effectively stratify patients into clinically meaningful phenotypes. This study integrates standardised clinical, demographic, anthropometric, and polysomnographic data from multiple sleep centres, providing the first large-scale Italian investigation specifically focused on the heterogeneity of OSA determined by hypoxaemia. This approach offers a comprehensive and real-world assessment of how nocturnal desaturation patterns contribute to phenotypic variability within the disorder.

This present work originates from the multicenter program “Living with OSA and CPAP: The Apulia Region Experience,” encompassing a collaborative network of sleep centers throughout Southern Italy. The project aims to provide a comprehensive picture of OSA in a real-world context, integrating clinical, demographic, polysomnographic, and therapeutic information.

The ultimate goal of the project is to improve understanding of OSA heterogeneity by delineating clinically meaningful phenotypes that account for differences in disease burden, comorbidities, and treatment response across diverse care settings. As first work, the Living with OSA and CPAP study aimed to phenotypically characterize patients with OSA using standardized clinical and polysomnographic data, with a particular emphasis on hypoxemia-related traits. By exploring the interplay between nocturnal oxygen desaturation patterns and clinical features, this study seeks to identify distinct hypoxemic profiles that may underlie interindividual variability in disease severity and potential treatment outcomes.

## 2. Methods

### 2.1. Study Design and Setting

This study is part of the multicenter project “Living with OSA and CPAP: The Apulia Region Experience”, involving a network of 15 sleep laboratories across the Apulia region (Southern Italy) and approved by the regional ethics committee (approval number: 2170/CEL). The project includes both retrospective and prospective arms.

A standardized case report form and structured interview were developed to collect harmonized data across centers, including information on referral type and reason, demographic, anthropometric data, bed partner observations and clinical characteristics including diurnal and nocturnal OSA symptoms, sleep–wake profile, coexistence of other sleep disorders, OSA treatment, therapies and other medical comorbidities. In the prospective phase, clinical and demographic data are collected at baseline, and at 3-month and 6-month follow-up visits. At each visit, patients also complete validated questionnaires assessing daytime sleepiness (Epworth Sleepiness Scale) [15], sleep quality (Pittsburgh Sleep Quality Index) [16], the presence of insomnia (Insomnia Severity Index) [17], and quality of life (36-Item Short Form Health Survey, SF-36) [18]. Polysomnographic data, as well as information on treatment (Auto-PAP, CPAP, or other), mask type, pressure settings, and supplemental oxygen use, are also collected. CPAP adherence data are recorded, including the reasons for treatment interruption.

The present analysis focuses exclusively on the retrospective arm and includes data extracted from a subset of centers that had completed standardized data extraction and harmonization at the time of analysis.

### 2.2. Data Source and Participants

**Figure 1:**
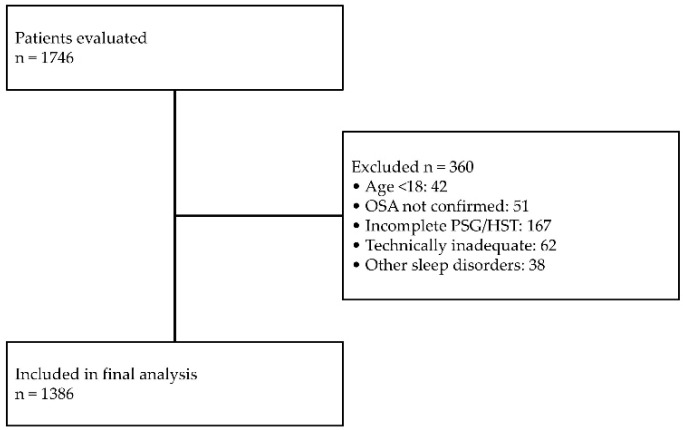
Flowchart of the study.

Data were retrospectively collected from electronic medical records of adult patients (≥18 years) referred to participating in sleep centers for suspected sleep-disordered breathing. Below is the flowchart of the study (Figure 1).

Inclusion Criteria

- Age ≥ 18 years
- Referral for suspected sleep-disordered breathing and confirmed diagnosis of OSA
- Completion of full-night PSG or home sleep testing (HST)
- Availability of standardized clinical and PSG/HST variables

Exclusion Criteria

- Age < 18 years
- Incomplete or missing PSG/HST data
- Technically inadequate or unreliable sleep recordings
- Presence of a primary sleep disorder other than sleep-disordered breathing (e.g., narcolepsy, idiopathic hypersomnia, parasomnias, circadian rhythm disorders) in the absence of SDB

Extracted variables included (i) demographic, anthropometric and lifestyle data: age, sex, body mass index (BMI), neck circumference smoking and alcohol habits, education, physical activity, menopause status; (ii) clinical data: nocturnal and diurnal associated OSA symptoms, comorbidities, medications, and Epworth Sleepiness Scale scores; (iii) PSG or HST parameters: time in bed, sleep onset and offset, percentage of supine and non-supine sleep, total and positional AHI (supine and non-supine), ODI, mean SpO_2_, minimum SpO_2_, maximum SpO_2_, and baseline SpO_2_, T90, total time SpO_2_ < 88% (T88%), and mean nocturnal nadir SpO_2_; and (iv) therapeutic data: CPAP or APAP prescription, CPAP pressure settings, supplemental oxygen, and ABG analysis when available.

All participating centers used standardized diagnostic criteria according to the 2021 American Academy of Sleep Medicine (AASM) guidelines [19].

### 2.3. Data Analyses

Descriptive analyses were conducted using SPSS software (version 26, IBM Corp., Armonk, NY, USA), while unsupervised analyses (clustering) were performed with Orange data mining software (version 3.36.2, Bioinformatics Laboratory, University of Ljubljana, Ljubljana, Slovenia).

HST data (AHI, ODI, T90, mean SpO_2_, nadir SpO_2_, mean minimum SpO_2_) were used to search for homogeneous groups of patients through hierarchical clustering. Hierarchical clustering was obtained after computing a matrix of distances among the data and selecting the most significant clusters from the resulting dendrogram. Distances were calculated using Ward’s method, a technique particularly suitable for biomedical datasets, in which the minimum variance criterion minimizes the total within-cluster variance. Finally, three clusters were identified, and the obtained clustering was assessed by means of silhouette index. Differences between clusters were determined by comparing the data: continuous variables with the Kruskal–Wallis test and categorical variables with the Chi-square test. Overall *p*-value corrections were performed using Bonferroni post hoc test. A *p*-value < 0.05 was considered statistically significant.

To explore the clinical applicability of the identified OSA phenotypes, we complemented the unsupervised clustering approach with a supervised predictive analysis aimed at estimating cluster membership using the available variables. An initial feature-ranking phase was conducted using multiple, complementary ranking methods (Gini index, χ^2^ test, ReliefF, and Fast Correlation-Based Filter). The concordance across different ranking algorithms was used to identify the most robust and informative predictors. Through this cross-ranking strategy, three variables consistently emerged as the top contributors to cluster discrimination: AHI, ODI and T90. These parameters were therefore selected for the predictive models, as they jointly capture both the frequency of respiratory events and the burden of nocturnal hypoxemia. Using this feature set, several supervised machine learning algorithms were trained and tested: CN2 rule induction, Naïve Bayes, Logistic Regression, Neural Network, kNN. Model performance was evaluated using a 5-fold cross-validation framework to ensure robustness and limit overfitting. The predictive performance of the supervised machine learning models was evaluated using multiple complementary metrics, including the area under the receiver operating characteristic curve (AUC), classification accuracy (CA), F1-score, precision, recall, and Matthews correlation coefficient (MCC).

## 3. Results

A total of 1386 patients were included in the study, divided into three distinct clusters (C1 = 606; C2 = 199; C3 = 581) according to sleep data.

### 3.1. Characterization of Clusters Based on Nocturnal Oximetry and AHI

**Figure 2:**
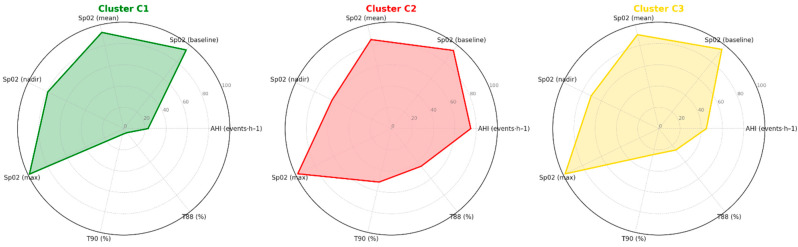
The radar charts show the nocturnal differences in terms of oximetry parameters and apnoea–hypopnoea index (AHI) in the three clusters.

Figure 2 provides an overview of the key nocturnal oximetry variables and AHI values contributing to the phenotypic separation across clusters.

Cluster C1 exhibited the most preserved oxygenation profile, with higher mean and nadir SpO_2_ values and minimal hypoxemia exposure. Cluster C2 showed the most profound alterations, including the lowest mean and nadir SpO_2_ and markedly elevated T90 and T88 values, consistent with severe OSA. Cluster C3 presented an intermediate pattern with moderate hypoxemia and AHI values between clusters C1 and C2.

### 3.2. Demographic and Clinical Characteristics

Table 1 summarizes the demographic, anthropometric, clinical, and comorbidity profiles of the three clusters.

The mean age was 62.2 ± 12.5 years, with significant differences among the groups: patients in cluster C2 were younger (59.4 ± 13.0 years), whereas those in cluster C3 were older (63.7 ± 11.8 years; *p* < 0.001). The overall prevalence of males was 71%, higher in cluster C3 (75%) compared to C1 (68%; *p* = 0.031). No differences were observed regarding menopausal status or education level. Smoking was more frequent in clusters C2 and C3 compared to C1 (72% and 74% vs. 60%; *p* = 0.017). Body mass index (BMI) was significantly higher in cluster C2 (39.2 ± 8.2 kg/m^2^) compared to C1 (30.9 ± 5.6 kg/m^2^) and C3 (34.9 ± 7.4 kg/m^2^; *p* < 0.001). Similarly, neck circumference was greater in C2 and C3 compared to C1 (*p* < 0.001).

Cluster C1 showed higher PaO_2_ values and lower PaCO_2_ values compared to C2 and C3 (*p* < 0.05). HCO_3_^−^ was higher in clusters C2 and C3 compared to C1 (*p* = 0.003).

Metabolic disorders were significantly more frequent in C2 (76%) and C3 (70%) compared to C1 (50%; *p* < 0.001). No relevant differences emerged for other comorbidities.

### 3.3. Sleep-Related Symptoms

The distribution of nocturnal and daytime symptoms is presented in Table 1. The frequency of snoring, choking, and nocturia was high and comparable among the groups. Daytime symptoms, however, showed marked differences: cluster C1 reported symptoms less frequently compared to the others (78% vs. 94% in C2 and 92% in C3; *p* < 0.001). Morning headache was more common in C2 (37% vs. 23% in C1 and C3; *p* = 0.008), whereas fatigue was reported less frequently in C1 (51% vs. 67% in C2 and 65% in C3; *p* = 0.001). ESS was higher in C2 (11.2 ± 5.6) compared to the other groups (*p* = 0.001).

### 3.4. Polysomnographic Data

The polysomnographic differences among clusters are detailed in Table 1. Time in bed and sleep/wake schedules did not differ significantly. However, the supine position was more frequent in C2, with a consequently higher AHI (74.9 ± 17.9 vs. 22.9 ± 10.5 in C1 and 44.8 ± 15.2 in C3; *p* < 0.001).

Cluster C2 showed the most severe respiratory disturbance compared to other clusters, with markedly elevated ODI values, prolonged hypoxemia as indicated by T90% and T88%, and the lowest mean and nadir SpO_2_ levels (*p* < 0.001). In contrast, Cluster C1 exhibited substantially milder alterations, with the highest mean and nadir SpO_2_ values and minimal time spent below 90% saturation, indicating preserved ventilatory efficiency and limited exposure to intermittent hypoxia. Cluster C3 also showed significant nocturnal hypoxemia, with a prolonged T90% and moderately reduced SpO_2_ values, although to a lesser extent than Cluster C2.

### 3.5. Therapeutic Features

Treatment-related characteristics are displayed in Table 1. The use of CPAP was higher in clusters C2 (69%) and C3 (70%) compared to C1 (39%; *p* < 0.001). Conversely, the use of auto-PAP was more frequent in C1 (59% vs. 45% in C2 and 34% in C3; *p* < 0.001). Treatment pressures were higher in C2, as was the need for supplemental oxygen (69% vs. 28% in C1 and 32% in C3; *p* = 0.005).

### 3.6. Phenotypic Summary of Clusters

**Figure 3:**
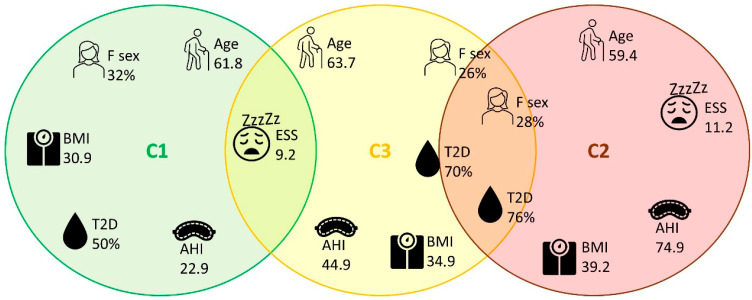
Schematic representation of the three clusters (C1, C2, and C3) identified. The variables located within or near each circle describe the predominant characteristics of the corresponding cluster. Variables positioned at the intersections indicate similar or overlapping values between adjacent groups, highlighting shared phenotypic traits.

Figure 3 offers a schematic visualization of the predominant and overlapping features characterizing each cluster. The clusters were defined as follows: C1 “mild–non-obese,” since patients were younger, with lower BMI and neck circumference values, less severe OSA, less hypoxemia, and a reduced metabolic burden; C2 “severe–obese–hypoxemic,” due to the presence of marked obesity, more severe OSA, significant hypoxemia, very frequent daytime symptoms, and higher metabolic comorbidity; C3 “older–comorbid,” as age was higher, with a male predominance, intermediate BMI, moderate-to-severe OSA and also cardiometabolic comorbidities.

Cluster prediction analysis showed that logistic regression and neural network models had the best overall performance, with almost identical results across all evaluation parameters. Both models achieved an AUC of 0.973, classification accuracy was 0.889, with equally high values for F1 score, precision and recall (all equal to 0.889). The robustness of the predictions was further supported by high MCC values (0.819 for the neural network and 0.818 for logistic regression), indicating a strong agreement between the predicted and actual cluster assignments. Overall, these results demonstrate that cluster membership can be reliably predicted using only three variables derived from the PSG (AHI, ODI, and T90).

## 4. Discussion

The study included a large, heterogeneous cohort recruited from multiple sleep laboratories, reflecting a real-world scenario in which diagnostic and therapeutic resources vary considerably across the centers. This study therefore captured the diversity of clinical practice, diagnostic resources, and referral patterns typical of non-tertiary centers.

We identified 3 reproducible OSA phenotypes defined by oximetric and respiratory parameters: Cluster 1, Mild–Non-Obese, characterized by lower AHI and limited nocturnal hypoxemia; Cluster 2, Severe–Obese–Hypoxemic, defined by extremely high AHI, severe hypoxemia (prolonged T90 and low SpO_2_ nadir), and a marked metabolic burden; and Cluster 3, Older–Comorbid, encompassing older patients with moderate-to-severe OSA and a high prevalence of cardiometabolic and systemic comorbidities. These phenotypes differed significantly across demographic, anthropometric, and oximetric parameters, as well as in therapeutic approaches (CPAP, APAP, and oxygen supplementation).

The 3 identified clusters aligned with recent literature studies that privilege metrics of hypoxia beyond traditional metrics of AHI to define OSA phenotypes [12,20,21]. OSA has long been recognized as a heterogeneous disorder with variable symptom profiles, comorbidities, and treatment responses despite similar AHI values [22,23]. Recent efforts have focused on phenotyping (based on clinical and polysomnographic profiles) and endotyping (based on underlying pathophysiology such as loop gain, arousal threshold, muscle responsiveness, and anatomical collapsibility) [22].

In the past two decades, increasing attention has been paid to the use of cluster analysis based on symptoms demographic associated comorbidities and polysomnographic findings in order to disentangle the phenotypic heterogeneity of OSA. Ye et al. [20] were among the first to apply cluster analysis to identify subtypes of patients with OSA and described three symptom-based clusters, i.e., minimally symptomatic, excessive daytime sleepiness, and disturbed sleep, highlighting that symptom expression and comorbidity, rather than AHI, define meaningful OSA subtypes. Bailly et al. identified distinct clusters among >18,000 patients, based on symptom profiles, anthropometrics, and comorbidities, revealing wide variation in disease burden and cardiovascular risk among patients with similar AHI values [24]. Ferreira-Santos et al. [25] confirmed the existence of three phenotypic clusters differentiated by gender, obesity, neck circumference, and alcohol use, again underscoring the heterogeneity of OSA even in smaller cohorts. Notably, gender differences also emerged from the ESADA database, with women showing distinct symptom profiles and comorbidity patterns compared to men, further highlighting the importance of sex-specific phenotyping [12,26].

Recent analyses have extended clustering beyond clinical features to include polysomnographic and oximetric data, as in the study by Zinchuk et al. [27] who used polysomnographic indices to identify seven phenotypes differing in sleep architecture, cooccurrence of other sleep disorders (e.g., Periodic limb movements) oxygen desaturation burden in NREM and REM sleep and found that this cluster assignment can predict independently adverse cardiovascular CVD or death.

Zhang et al. [28] conducted a multidimensional cluster analysis in a well-characterized Asian cohort of patients with moderate-to-severe OSA, integrating clinical symptoms, anthropometric indices, polysomnographic data, craniofacial morphology, and CPAP titration response.

The Icelandic Sleep Apnea Cohort [29] provided further evidence that PAP adherence and treatment response vary significantly by phenotype, particularly between the “sleepy” and “disturbed sleep” clusters. Furthermore, comorbidities have an important influence on long-term CPAP continuation in patients with OSA as demonstrated by the study by Pépin [30].

Recent cluster-based and machine learning studies have further explored the prognostic implications of OSA phenotypes. Kim et al. [31] applied an unsupervised clustering approach and identified four distinct OSA phenotypes, demonstrating that the cluster characterized by severe nocturnal hypoxemia and sleep fragmentation had significantly higher 10-year cardiovascular and all-cause mortality, independent of conventional AHI severity. Similarly, Labarca et al. [11] and Silveira et al. [32] found that clusters defined by prolonged nocturnal desaturation and metabolic comorbidities were independently associated with increased cardiovascular risk and 5-year mortality. More recently, Tondo et al. [13] applied an unsupervised machine learning approach to refine long-term mortality risk stratification in OSA, identifying distinct phenotypes characterized by varying degrees of nocturnal hypoxemic burden and comorbidity profiles. Across models, nocturnal hypoxemia consistently emerged as the strongest independent predictor of mortality.

In line with previous studies [11,13,28,33], our unsupervised clustering analysis based mainly on oximetric variables, supports the physiological and prognostic relevance of hypoxemia patterns in distinguishing OSA subtypes. Moving from the traditional severity indices such as AHI that fail to capture the true physiological burden of the disease emerging evidence have identified nocturnal hypoxic burden (HB) which integrates the depth, duration, and frequency of desaturations, as a more robust predictor of cardiovascular, metabolic, and neurocognitive outcomes [33,34].

The “severe–obese–hypoxemic” phenotype represent the “high hypoxic burden” endotype [22]. Physiologically, chronic intermittent hypoxia (IH) triggers a cascade of maladaptive responses including sympathetic hyperactivation, oxidative stress, endothelial dysfunction, and systemic inflammation [23]. The subsequent cascade of events that follow explain the association with OSA related complications including the well-known cardiovascular complication such as resistant hypertension [35]. Furthermore, IH and sleep fragmentation also induce metabolic dysregulation, impairing insulin sensitivity, promoting adipose inflammation, and increasing leptin resistance which contribute to the bidirectional link between OSA and obesity/metabolic syndrome [36]. Notably, this group required higher fixed pressures and more frequent supplemental oxygen, consistent with the reduced upper-airway responsiveness and greater ventilatory load observed in severe OSA phenotypes. This cluster thus represents the subgroup at highest cardiometabolic and mortality risk. Our data confirm that this “severe obese-hypoxemic” phenotype remains the archetypal high-risk endotype of OSA, representing a key target for early detection and aggressive treatment.

On the contrary, cluster 1 represents patients with lower AHI, minimal hypoxemia, and a limited burden of comorbidities [22]. These patients, often less obese, likely display OSA driven primarily by anatomical factors (e.g., craniofacial structure or positional dependency) rather than systemic pathophysiological dysregulation. This phenotype is similar to “low hypoxic burden” or “non-obese positional” or “non-obese mild” phenotypes described previously [37] who also noted milder cardiovascular risk profiles and better treatment adherence. Their risk of end-organ complications appears lower. Clinically, this group may benefit from early lifestyle interventions and positional therapy or oral appliance therapy rather than immediate long term CPAP use [37].

The “Older–Comorbid” phenotype (Cluster 3) identified in our study includes patients with advanced age, moderate-to-severe OSA, and a substantial burden of cardiometabolic and systemic comorbidities. Although these individuals exhibited less profound nocturnal hypoxemia than those in the “Severe–Obese–Hypoxemic” group, the interaction between aging-related neural vulnerability and chronic sleep fragmentation may have important neurocognitive implications.

A growing body of evidence indicates that OSA is associated with cognitive impairment [38,39,40]. Mechanistically, intermittent hypoxia and sleep fragmentation lead to oxidative stress, cerebrovascular dysfunction, and impaired clearance of neurotoxic metabolites the glymphatic system, processes that accelerate neurodegenerative trajectories [38,39,40].

In elderly individuals, even mild-to-moderate OSA may exacerbate cognitive vulnerability due to reduced neuroplasticity and cumulative vascular burden [41,42,43]. Longitudinal data suggest that OSA-related hypoxemia is independently associated with increased risk of mild cognitive impairment and dementia [44,45]. Chronic intermittent hypoxia can promote β-amyloid aggregation and tau phosphorylation, linking OSA pathophysiology to Alzheimer-type neurodegeneration [46,47]. Literature data have shown that patients with untreated OSA exhibit a faster rate of cognitive decline and a higher risk of developing mild cognitive impairment and AD compared to age-matched controls [41,42,43]. Furthermore, patients with AD exhibit a markedly higher prevalence of OSA than cognitively normal individuals, suggesting a bidirectional relationship in which sleep-disordered breathing exacerbates amyloid-β and tau pathology through intermittent hypoxia, sleep fragmentation, and impaired glymphatic clearance [41,42,43]. Structural and functional neuroimaging studies have also demonstrated reduced gray matter volume and white matter integrity in key cognitive networks such as the hippocampus, prefrontal cortex, and cingulum in patients with OSA [48].

In this context, the “Older–Comorbid” phenotype identified in our study may represent a particularly vulnerable subgroup of patients who are likely to experience cumulative exposure to intermittent hypoxia and sleep disruption over time, which may amplify neurodegenerative processes.

The overlap between vascular risk factors, metabolic dysfunction, and hypoxemia observed in this cluster could synergistically impair cerebrovascular regulation and glymphatic clearance, thereby fostering amyloid accumulation and neuronal injury. Within this context, the phenotype identified in our cohort likely represents a subgroup particularly susceptible to neurocognitive decline, even in the absence of extreme hypoxemia. Recognition of this phenotype highlights the need for a comprehensive, multidisciplinary assessment in elderly OSA patients including systematic cognitive screening. Early recognition and effective management of OSA in this population could mitigate neurocognitive decline, delay progression toward dementia, and ultimately improve functional outcomes and quality of life.

Overall, the three phenotypes identified in our study capture the multidimensional nature of OSA. Our findings reinforce the growing recognition that OSA severity cannot be adequately defined by AHI alone, but rather by the pattern and physiological consequences of nocturnal hypoxemia. Identifying such phenotypes provides an opportunity to refine risk stratification and personalized management, ranging from lifestyle interventions and weight optimization to tailored CPAP titration and multimodal control of cardiometabolic and neurocognitive comorbidities.

Importantly, our study represents one of the first multicenter, real-world analyses from Southern Italy, encompassing both urban and rural sleep centers where diagnostic awareness, referral pathways, and technical resources may vary. Despite these differences, consistent and clinically meaningful clustering patterns emerged, underscoring the robustness and reproducibility of data-driven phenotyping even in non-tertiary clinical settings.

This geographical and organizational diversity enhances the external validity of our findings and supports the broader applicability of phenotype-based approaches to OSA management. Ultimately, integrating oximetry-derived metrics and phenotypic clustering into clinical practice may bridge the gap between conventional AHI-based classification and precision medicine, paving the way for more targeted and outcome-oriented care for patients living with OSA.

Limitations of the Study: It is necessary to acknowledge certain limitations that may influence the interpretation of the results. The retrospective design does not allow for complete control over the completeness and accuracy of the clinical information collected. Although the multicentre approach increases generalisability, differences in diagnostic equipment, scoring procedures and local organisation may have introduced variability, despite the adoption of standardised data harmonisation protocols.

The analysis is based exclusively on cross-sectional baseline data and therefore does not allow for the evaluation of longitudinal outcomes or causal relationships. The prognostic significance of the identified phenotypes therefore remains to be clarified, especially in relation to cardiovascular, metabolic, and neurocognitive pathways. Finally, the cohort comes from a single geographical area, which may limit the applicability of the results to populations with different genetic, environmental, or healthcare systems.

Recommendations for Future Research: Future studies should aim to prospectively validate the identified phenotypes, preferably in large multicentre cohorts using uniform diagnostic protocols and long-term follow-up. This would allow the prognostic significance of each phenotype to be defined, particularly with regard to cardiometabolic outcomes, neurocognitive decline and mortality. The integration of pathophysiological endotypes, such as loop gain, arousal threshold, upper airway collapsibility, and muscle responsiveness, could improve biological interpretation and further refine phenotypic classification.

It will also be important to explore phenotype-specific therapeutic strategies, including personalised PAP titration methods, alternative treatments in subjects who are not eligible for PAP, and integrated approaches to comorbidity management. Finally, the application of advanced analytical methods, such as machine learning algorithms on larger and more diverse populations, may contribute to the development of operational tools useful for risk stratification, outcome prediction, and treatment personalisation in clinical practice.

## 5. Conclusions

The study suggests that the use of oximetric parameters allows for the clear distinction of three clinical profiles of OSA within a large Italian cohort observed in real-world practice. The three phenotypes identified have different demographic, metabolic and respiratory characteristics and show that the burden of nocturnal hypoxaemia is a more informative discriminating factor than AHI alone.

The identification of these phenotypes highlights the potential value of a phenotypic approach in the characterisation of OSA and supports the hypothesis of a future evolution towards more personalised management strategies. However, in the absence of longitudinal data, the prognostic implications and impact on therapeutic decisions cannot be directly inferred from this study and require confirmation in dedicated prospective studies with long-term follow-up.

## Figures and Tables

**Table 1:** Population characteristics and comparison between groups.

Variables	Total	C1	C2	C3	Overall *p*
	(*n* = 1386)	(*n* = 606)	(*n* = 199)	(*n* = 581)	
Age (years)	62.2 ± 12.5	61.8 ± 12.8 (§)	59.4 ± 13.0 (¶)	63.7 ± 11.8 (§¶)	<0.001
Gender (% male)	71%	68% (§)	69%	75% (§)	0.031
Menopause (% Yes)	34%	31%	38%	37%	0.721
Smoking habits (% Yes)	68%	60% (§)	72%	74% (§)	0.017
Alcohol (% Yes)	34%	35%	33%	33%	0.958
Education (years)	11.1 ± 4.7	12.5 ± 5.1	11.5 ± 4.1	10.2 ± 4.7	0.057
BMI (kg·m^−2^)	33.8 ± 7.4	30.9 ± 5.6 (¤§)	39.2 ± 8.2 (¤¶)	34.9 ± 7.4 (§¶)	<0.001
Neck circumference (cm)	43.4 ± 4.8	41.5 ± 4.7 (¤§)	45.6 ± 5.0 (¤¶)	44.1 ± 4.2 (§¶)	<0.001
Physical activity (% Yes)	6%	3%	7%	6%	0.787
*Nocturnal Symptoms*					
Snoring (% Yes)	89%	86%	91%	92%	0.078
Choking (% Yes)	53%	52%	59%	52%	0.477
Nocturia (% Yes)	62%	62%	65%	62%	0.818
Sweating (% Yes)	31%	34%	31%	29%	0.768
Involuntary movements (% Yes)	21%	27%	23%	15%	0.121
*Diurnal Symptoms*	88%	78% (¤§)	94% (¤)	92% (§)	<0.001
EDS (% Yes)	68%	62%	74%	72%	0.177
Morning headache (% Yes)	26%	23% (¤)	37% (¤¶)	23% (¶)	0.008
Dry mouth (% Yes)	65%	60%	69%	67%	0.247
Mental confusion (% Yes)	22%	28%	22%	18%	0.284
Memory disturbances (% Yes)	42%	46%	38%	41%	0.392
Attention impairment (% Yes)	40%	44%	34%	39%	0.256
Fatigue (% Yes)	61%	51% (¤§)	67% (¤)	65% (§)	0.001
Other cognitive symptoms (% Yes)	37%	39%	47%	32%	0.062
Sleep habit modification (% Yes)	7%	9%	4%	8%	0.642
*Comorbidities*					
Respiratory comorbidities (% Yes)	21%	19%	14%	25%	0.075
Cardiovascular comorbidities (% Yes)	73%	71%	72%	74%	0.713
Internal medicine comorbidities (% Yes)	52%	49%	52%	54%	0.599
Metabolic comorbidities (% Yes)	65%	50% (¤§)	76% (¤)	70% (§)	<0.001
Neurological comorbidities (% Yes)	19%	21%	12%	20%	0.222
Arterial Blood Gas analysis					
PaO_2_ (mmHg)	79.1 ± 12.5	84.1 ± 11.6 (¤§)	76.1 ± 12.9 (¤)	77.3 ± 12.1 (§)	<0.001
PaCO_2_ (mmHg)	41.1 ± 6.7	39.9 ± 3.6 (¤)	42.4 ± 6.8 (¤)	41.3 ± 7.9	0.041
pH	7.4 ± 0.0	7.4 ± 0.0 (§)	7.4 ± 0.0	7.4 ± 0.0 (§)	0.021
SpO_2_ (%)	97.4 ± 1.7	97.7 ± 1.8	97.4 ± 1.4	97.3 ± 1.8	0.523
HCO_3_^−^ (mmol·L^−1^)	25.9 ± 2.7	25.2 ± 2.1 (¤§)	26.5 ± 3.1 (¤)	26,1 ± 2.8 (§)	0.003
*Sleep study data*					
TIB	6.8 ± 1.3	6.7 ± 1.3	6.8 ± 1.2	6.9 ± 1.3	0.646
Sleep onset time	22.1 ± 3.7	22.7 ± 0.8	21.2 ± 5.7	22.4 ± 2.7	0.165
Sleep offset time	5.8 ± 1.1	5.8 ± 1.1	5.9 ± 1.2	5.8 ± 1.2	0.644
TIB supine %	49.6 ± 29.5	46.6 ± 28.2 (¤)	59.8 ± 30.4 (¤¶)	49.4 ± 30.0 (¶)	<0.001
TIB non-supine %	49.2 ± 29.3	51.7 ± 28.1 (¤)	41.5 ± 30.3 (¤¶)	48.9 ± 30.1 (¶)	0.002
AHI (events·h^−1^)	39.4 ± 22.5	22.9 ± 10.5 (¤§)	74.9 ± 17.9 (¤¶)	44.8 ± 15.2 (§¶)	<0.001
AHI supine (events·h^−1^)	44.2 ± 25.8	34.0 ± 20.2 (¤§)	72.5 ± 25.5 (¤¶)	46.7 ± 23.7 (§¶)	<0.001
AHI non-supine (events·h^−1^)	28.0 ± 27.2	15.7 ± 14.0 (¤§)	61.9 ± 36.1 (¤¶)	31.2 ± 23.6 (§¶)	<0.001
ODI (events·h^−1^)	38.8 ± 23.1	21.5 ± 10.1 (¤§)	75.2 ± 18.5	44.6 ± 15.4 (§¶)	<0.001
Baseline SpO_2_%	94.7 ± 4.4	94.5 ± 3.9	93.9 ± 5.9	95.2 ± 4.2	0.191
Mean SpO_2_%	90.9 ± 3.7	92.7 ± 2.0 (¤§)	85.8 ± 4.9 (¤¶)	90.5 ± 2.6 (§¶)	<0.001
Minimum SpO_2_%	73.9 ± 10.3	79.4 ± 7.3 (¤§)	61.9 ± 9.9 (¤¶)	70.8 ± 8.7 (§¶)	<0.001
Maximum SpO_2_%	98.2 ± 2.9	98.6 ± 2.1	97.8 ± 2.7	98.0 ± 3.6	0.236
T90%	20.4 ± 24.5	5.6 ± 7.6 (¤§)	51.5 ± 28.2 (¤¶)	25.1 ± 22.4 (§¶)	<0.001
T88%	16.6 ± 23.5	4.9 ± 8.0 (¤§)	45.2 ± 29.8 (¤¶)	25.7 ± 24.8 (§¶)	<0.001
Mean minimum SpO_2_	86.5 ± 5.3	87.7 ± 5.3 (¤)	81.9 ± 6.7 (¤¶)	87.1 ± 3.4 (¶)	<0.001
CPAP	59%	39% (¤§)	69% (¤)	70% (§)	<0.001
A-PAP	44%	59% (¤§)	45% (¤)	34% (§)	<0.001
Fixed pressure setting	9.7 ± 2.4	8.8 ± 1.7 (¤)	10.9 ± 2.7 (¤)	9.8 ± 2.3	<0.001
Mask type	49%	38% (¤§)	58% (¤)	53% (§)	<0.001
Supplemental oxygen	33%	28% (¤)	69% (¤¶)	32% (¶)	0.005
Baseline ESS score	9.5 ± 5.2	9.2 ± 5.1 (¤)	11.2 ± 5.6 (¤¶)	9.2 ± 5.1 (¶)	0.001

Data are expressed as mean ± standard deviation for continuous variables and percentage for categorical variables. Statistical significance between the three subgroups after Bonferroni correction: ¤ C1 vs. C2; § C1 vs. C3; ¶ C2 vs. C3. *Abbreviations:* AHI: Apnea/Hypopnea Index; A-PAP: Auto-titrating Positive Airway Pressure; BMI: Body Mass Index; CPAP: Continuous Positive Airway Pressure; EDS: Excessive Daytime sleepiness; ESS: Epworth Sleepiness Scale; HCO_3_^−^: bicarbonate; ODI: Oxygen desaturation Index; PaO_2_: arterial partial pressure of oxygen; PaCO_2_: arterial partial pressure of carbon dioxide; TIB: Time in Bed; T88% Cumulative time spent with SpO_2_ < 88%; T90%: Cumulative time spent with SpO_2_ < 90%.

## Data Availability

No new data were created or analyzed in this study.
